# Use of Flow Cytometric Analysis to Examine the Uptake of Apoptotic Bodies by Healthy Hepatocytes

**DOI:** 10.1186/1476-5926-2-S1-S40

**Published:** 2004-01-14

**Authors:** Carol A Casey, Cheryl R Baldwin, Jacy L Kubik, Agnes M Hindemith, Benita L McVicker

**Affiliations:** 1 Liver Study Unit, VA Medical Center and University of Nebraska Medical Center, Omaha, NE, USA

## Introduction

The spectrum of liver disease after alcohol abuse is similar to many other types of liver diseases in that damage to the hepatocyte, or parenchymal cell, is the cardinal event in the pathogenesis of injury. Damage to the liver, however, involves not only the hepatocyte but also the nonparenchymal cells: the Kupffer, endothelial, and stellate cells which release inflammatory and fibrogenic factors (such as cytokines and chemokines) that lead to altered liver pathology. We have studied extensively the effects of ethanol administration on protein trafficking in the liver, focusing most of our work on the hepatocyte and the process of endocytosis by these cells, using the endocytic pathway of the asialoglycoprotein receptor (ASGP-R) as a model [1-8]. Currently in our laboratory we are examining the effect of ethanol administration on the process of apoptosis (programmed cell death) to determine a role for altered endocytosis in this process. Apoptosis is recognized not only as one of the initiating events in toxic liver injury, but is also increasingly recognized as a key mechanism in tissue inflammation and fibrosis [9,10]. Of interest to us is work showing that healthy hepatocytes can bind and internalize apoptotic bodies [11,12], and that the hepatocellular ASGP-R is involved in this clearance. We have utilized an *in vitro *system to characterize uptake of apoptotic bodies in isolated hepatocytes and define a role for the ASGP-R in this uptake. Our goal is to determine if there is an impaired ability to take up bodies in hepatocytes from ethanol-fed animals compared with control hepatocytes. An ethanol-induced impairment in the ability to remove apoptotic cells in the liver parenchyma could lead to an accumulation of bodies thereby disturbing the hepatic architecture and contributing to the initiation of fibrosis, specifically by activation of the non-parenchymal cell population. The work described in this present study was performed to characterize the *in vitro *system using flow cytometry to examine uptake of apoptotic bodies by healthy hepatocytes. Our long-term goal is to determine whether inadequate removal of apoptotic cells, presumably via altered receptor-mediated endocytosis (RME), plays a role in the course of pathogenesis of alcoholic liver injury.

## Methods

### Hepatocyte Preparation

Hepatocytes were obtained from male Wistar rats using a collagenase perfusion method that is routinely performed in our laboratory [1-8]. Ethanol feeding was performed using Lieber-DeCarli diet as described previously [1-8].

### Preparation of apoptotic H4 cells

Rat hepatoma H4IIE cells were cultured in William's E media. Apoptosis was induced in sub-confluent cells in the presence of 10 mg cisplatin/ml for 20–24 hours at 37 degrees C.

### Labeling and characterization of target apoptotic cells for phagocytosis assays

The generated apoptotic cells were labeled according to the manufacturer's (Sigma) protocol with the fluorescent probe, 5–6-carboxytetramethyl-rhodamine-succinimidyl-ester (TAMRA). The extent and characteristics of apoptotic cells obtained by the cisplatin treatment was determined by Hoechst 33342 microscopic evaluation, Annexin V-FITC staining, viability staining (trypan blue) and ethidium bromide and acridine orange (EB/AO; to distinguish between viable, apoptotic, and necrotic cells) and lectin binding: all were performed by standard procedures.

### Phagocytosis assays

TAMRA-labeled apoptotic or non-apoptotic H4 cells (5–10 million) were incubated with freshly isolated rat hepatocytes (3–5 million) in the presence or absence of 200 mM N-acetylgalactosamine and galactose (GalNac/Gal), 200 mM glucose (Glu) or anti-ASGP-R antibody or non-immune serum (1:50 dilution, which is approximately 0.8–1.1 mg/ml protein final in the assay). To quantify the phagocytosis of apoptotic cells, we employed a flow cytometric method. Briefly, after incubation of the fluorescently labeled apoptotic or non-apoptotic cells with isolated rat hepatocytes cells at 37 degrees C, cell aliquots were analyzed by flow cytometry using the FACSCalibur (Becton Dickinson) with emission for the TAMRA-labeled cells. Fluorescence-activated cell sorting of TAMRA-positive rat hepatocytes was performed using a FACStarPLUS system (Becton Dickinson) with emission collected using a 575/26-band pass filter. Identification of the phagocytosed apoptotic cells was verified through confocal microscopy of sorted cells. The cells (approximately 1.0 – 10^5^) were cytospun onto glass slides, stained with Wright-Giemsa stain, and examined using a confocal laser-scanning microscope (Carl Zeiss LSM 410 invert with an argon-krypton laser with DIC capabilities) at the appropriate wavelengths.

## Results and Discussion

We initially performed ligand internalization studies with freshly isolated hepatocytes to establish that the endocytosis results were representative of the previous experiments we performed for protein trafficking studies in our laboratory [1-8]. We then used these cells in combination with apoptotic bodies obtained from H4 cells treated with cisplatin, as described in the "Methods" section. Incubation of the hepatocytes with the labeled bodies followed by flow cytometric analysis showed that the hepatocytes were capable of uptake of apoptotic H4 cells over a 60 minute time course. In addition, the phagocytosis of the apoptotic cells was shown to be significantly impaired (40–80%) in the presence of the polyclonal antibody specific for ASGP-R as well as the introduction of competing sugars (GalNac/Gal) in the media. The data from this *in vitro *assay indicates that the ASGP-R is involved in the recognition and uptake of apoptotic cells and suggests an association with alterations in clearance of cells by the ASGP-R. Confirmation of apoptotic cell uptake by the rat hepatocytes was performed by fluorescent microscopy analysis of sorted hepatocytes after flow cytometric analysis as well as by confocal microscopy. We feel that this experimental design provides an excellent means to examine apoptotic cell uptake in isolated cell populations. We will utilize this technique with hepatocytes from ethanol-fed animals to determine if there is an impaired uptake after ethanol administration, due to impaired RME by the ASGP-R. In future studies, we will also examine the time course for these impairments as well as a zonal distribution of the effects, since we have previously shown that altered RME is seen first and foremost in the perivenular region of the liver [4]. In addition, we plan to utilize this technique to measure uptake by the non-parenchymal cell populations, which are important in the phagocytic process.
